# Spatial and temporal variability in summer diet of gray wolves (*Canis lupus*) in the Greater Yellowstone Ecosystem

**DOI:** 10.1093/jmammal/gyab060

**Published:** 2021-05-29

**Authors:** Hanna K Lodberg-Holm, Bonnie S Teglas, Daniel B Tyers, Michael D Jimenez, Douglas W Smith

**Affiliations:** 1 Faculty of Technology, Natural Sciences and Maritime Sciences, Department of Natural Sciences and Environmental Health, University of South-Eastern Norway, P.O. Box 5003, NO-3800 Bø, Norway; 2 Department of Biology, University of Nevada, Reno, NV 89557, USA; 3 US Forest Service, Interagency Grizzly Bear Study Team, Northern Rockies Science Center, Bozeman, MT 59715, USA; 4 Post Office Box 177, Big Arm, MT 59910, USA; 5 Yellowstone Wolf Project, Yellowstone Center for Resources, P.O. Box 168, Yellowstone National Park, WY 82190, USA

**Keywords:** *Canis lupus*, diet, scat analysis, spatial, temporal, wolves

## Abstract

The role of predation by large carnivores in suppressing prey populations and structuring ecosystems is highly debated, calling for a detailed understanding of carnivore diets. Wolves (*Canis lupus*) roam across three continents and persist throughout widely different ecosystems. Their diet is flexible and may vary spatially as well as seasonally, which requires analysis of diet on different spatial and temporal scales. Few studies have investigated the summer diet of wolves, which is more variable, consists of smaller prey, and requires different methods than studying their winter diet. To better understand the summer diet of wolves, we combined three independently collected wolf scat data sets from three distinctly different portions of the Greater Yellowstone Ecosystem: Yellowstone National Park (2009), Grand Teton National Park (2003 – 2009), and the Absaroka-Beartooth Wilderness (2009 – 2010). These areas represent different ecological conditions and management regimes, which may impact wolf diet. We estimated relative biomass and compared occurrence of different prey species among packs, years, as well as the three regions. In total, we analyzed 1,906 wolf scats and found that neonate cervids, adult elk, and adult deer were the most important prey species in the summer diet of the wolves. We found dietary variation among packs residing in the same area, as well as across years. The occurrence of neonate cervids displayed the most variation, and low occurrence of this prey type often was associated with a more diverse diet. Wolf packs within the national parks had a higher occurrence of medium-sized prey (~ 50 – 70 kg) and lower occurrence of small-sized prey (≤ 20 kg) compared to wolves in the Absaroka-Beartooth Wilderness. These results demonstrate flexibility in summer diet across packs, years, and between regions within the Greater Yellowstone Ecosystem.

Predation is an important ecological function that affects the structure of ecosystems through direct and indirect effects (Estes et al. 2011; Ripple et al. 2014a). Predation may impact animal populations directly by suppressing prey species and mesopredators, which may have cascading effects through ecosystems (Prugh et al. 2009; Estes et al. 2011). One of the most famous examples of the complexity of predation is the reintroduction of wolves (*Canis lupus*) to Yellowstone National Park (1995 – 1997—Fritts et al. 1997; Smith et al. 2003), which sparked a controversial scientific debate on wolf influence on prey populations and behavior, and effects on the surrounding ecosystem (Kauffman et al. 2010; Ripple et al. 2014b; Painter et al. 2015; Boyce 2018). Wolves now are distributed across the Greater Yellowstone Ecosystem, which includes various subsystems with different compositions of prey species (Smith et al. 2004). Wolf diets often differ across ecosystems in response to prey density (Nowak et al. 2011), prey vulnerability, wolf colonization patterns (Capitani et al. 2004), and access to livestock (Meriggi et al. 1996). Intensive study efforts on wolf predation have been carried out in Yellowstone National Park to explore the direct and indirect effects of wolf predation on ungulate populations within the park, as well as wider ecosystem effects (Mech et al. 2001; Smith et al. 2004; Fortin et al. 2005; Vucetich et al. 2005; Metz et al. 2011, 2012). However, the majority of studies on wolf diet have been carried out in winter (Smith et al. 2004; Woodruff and Jimenez 2019), while investigations of summer diet are more scarce, and summer predation patterns therefore less understood (Metz et al. 2012).

Wolf dietary studies in summer face challenges of tracking wolf movements without the advantage of snow cover. Moreover, in the summer, wolf packs tend to be less cohesive, consume smaller prey items (Stahler et al. 2006; Jimenez et al. 2007; Metz et al. 2011), and have increased nighttime activity compared to winter (Peterson et al. 1984). Summer is a challenging season for wolves due to adult herbivores being less vulnerable to predation. In the winter, wolves can move more easily through the snow, but in the summer, that advantage is gone, and their prey generally are in better physical condition (Peterson and Ciucci 2003; Metz et al. 2012). In addition, movement of wolves in summer can be limited because wolves are tied to dens and rendezvous sites while they are rearing pups (Mech and Boitani 2003). However, the use of den and rendezvous sites in summer allows for relatively easy collection of wolf scats once the wolves disperse from these sites. Scat analysis is a valuable tool to study wolf diet because it may identify smaller prey species that may be characteristic of wolf summer diets. Frequency of occurrence and relative biomass consumed can be estimated from scats (Ciucci et al. 1996; Klare et al. 2011), and may contribute insight as to the importance of different prey species in the summer diet of wolves.

Our goal is to improve our understanding of wolf summer diet, because most research into wolf diet in the Greater Yellowstone Ecosystem has been undertaken in winter in specific areas within the national parks. Thus far, no study has examined wolf diet across a broader area within the Greater Yellowstone Ecosystem during summer, a time of year when ungulates and other prey are broadly distributed. To better address this objective, we broadened our scope and included national forest lands of the Absaroka-Beartooth Wilderness located north of Yellowstone National Park, which has different management regime than the national parks, but the least data on ungulates and wolves (Rickbeil et al. 2019), as well as Grand Teton National Park, which is located south of Yellowstone National Park.

We hypothesized that: 1) large-ungulate prey are less vulnerable during summer throughout the Greater Yellowstone Ecosystem, and predicted high occurrence of small- and medium-sized prey in the wolf diet; 2) wolf summer diet is highly opportunistic and adapted to spatial and temporal prey availability, which should be reflected by differing prey occurrence among wolf packs and across years; and 3) regional differences in wolf diet due to differing management regimes where wolves within the national parks are predicted to utilize larger prey than wolves in Absaroka-Beartooth Wilderness.

## Materials and Methods

### Study area

**Fig. 1.**
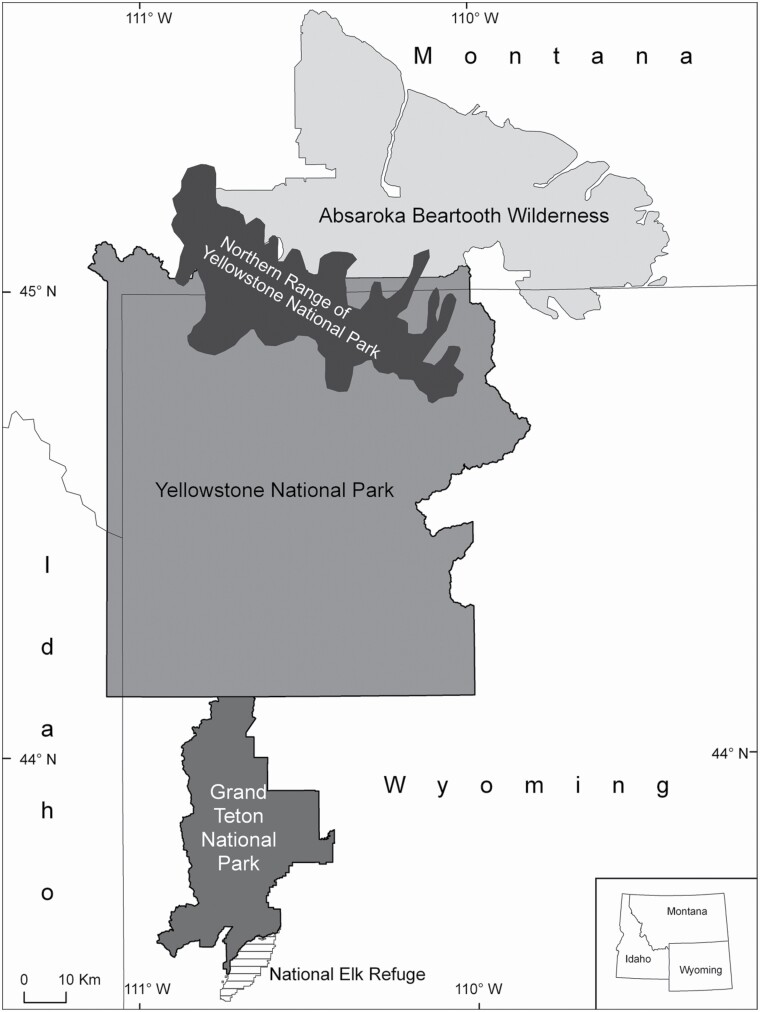
Map of the study area where wolf (*Canis lupus*) scats were collected including the northern range of Yellowstone National Park, Grand Teton National Park, and the Absaroka-Beartooth Wilderness.

The Greater Yellowstone Ecosystem (58,026 km^2^) consists of mostly public land in Montana, Wyoming, and Idaho, United States (Fig. 1). The area contains two national parks, Yellowstone National Park and Grand Teton National Park, which are protected against hunting, grazing, and resource extraction activities. The national parks are surrounded by national forests where state-regulated hunting seasons occur, such as the Absaroka-Beartooth Wilderness. Grand Teton National Park (1,356 km^2^) is characterized as a high valley surrounded by mountains with elevations between 1,900 and 3,900 m. The Absaroka-Beartooth Wilderness (3,818 km^2^) is located north of Yellowstone National Park in south-central Montana and northern Wyoming. In contrast to the two parks, state-regulated ungulate hunting occurs in the Absaroka-Beartooth Wilderness. The area is characterized by high mountain ranges with peaks to 3,600 m, tundra plateaus ranging from 1,500 to 3,000 m above sea level, and forested drainages going down to lower elevation. Yellowstone National Park (8,991 km^2^) is divided into two ecological subsystems, the Northern Range (1,000 km^2^) and the remaining park area. All sampling was carried out in the Northern Range, where elevation varies from 1,500 to 2,500 m. The Greater Yellowstone Ecosystem elk population (*Cervus canadensis*), the most common prey species for wolves in the area, seasonally migrate to the boundaries of the ecosystem, including the Northern Range (Smith et al. 2004; White et al. 2010). The Greater Yellowstone Ecosystem supports a variety of other potential prey species for wolves, such as bison (*Bison bison*), moose (*Alces alces*), bighorn sheep (*Ovis canadensis*), mule deer (*Odocoileus hemionus*), white-tailed deer (*O. virginianus*), pronghorn (*Antilocapra americana*), and beaver (*Castor canadensis*—Smith et al. 2004; Stahler et al. 2006). Overall, and although challenging due to the large area, ungulate distribution and abundance across the Greater Yellowstone Ecosystem is mostly known and routinely monitored by state and federal agencies due to the importance of ungulate hunting across the region (Metz et al. 2012; Rickbeil et al. 2019; Woodruff and Jimenez 2019). Large-ungulate hunting occurs within the Absaroka-Beartooth Wilderness (Ruth et al. 2003), which is not permitted within the two national parks, and may influence local ungulate densities. Still, prey availability on the local scale of each wolf pack is unknown. Other large carnivores found in the area include grizzly bears (*Ursus arctos*), black bears (*U. americanus*), cougars (*Puma concolor*), and coyotes (*C. latrans*—Yellowstone National Park 2015). Cattle (*Bos taurus*) grazing occurred on one allotment within Grand Teton National Park during the time of data collection (Trejo 2012). Cattle grazing also was permitted on a Forest Service allotment near the southeast corner of the Absaroka-Beartooth Wilderness, and cattle may stray into the wilderness areas. No domestic sheep (*O. aries*) are present within the study area.

### Scat collection

Wolf scats were collected from Grand Teton National Park and the National Elk Refuge, collectively referred to here as Teton, the Northern Range of Yellowstone National Park, hereafter referred to as Yellowstone, as well as the Absaroka-Beartooth Wilderness, referred to as Absaroka (Fig. 1). In Yellowstone, adult and pup scats were collected from wolf dens and rendezvous sites (hereafter “wolf homesites”) after wolves abandoned these areas in August of 2009. Adult wolf scats also were collected opportunistically from May to July 2009 at carcass sites, along trails, dirt roads, and at GPS-telemetry locations. To reduce potential pseudoreplication, opportunistically collected scats found grouped together (typically at a carcass site) were processed separately but the results (presence of prey items) were pooled together and treated as one sample unit for statistical analysis. This opportunistic collection only included scats with a diameter of ≥ 30 mm to exclude potential coyote scats (Weaver and Fritts 1979; Arjo et al. 2002). In Teton, adult and pup scats were collected at homesites after abandonment by the wolves in August and September during the years 2003–2009. Scats of all sizes were collected within the home areas to include pup scats, following the assumption that coyotes would not use habitat in close proximity to wolf homesites (Arjo et al. 2002; Derbridge et al. 2012). Scats in Absaroka were collected opportunistically along Forest Service maintained trails, which we used as transect routes to systematically search for scats between May and October in 2009 and 2010. Scats were assigned to different packs in Teton and Yellowstone based on annual monitoring of pack ranges, which tend to be relatively stable across years (Cassidy et al. 2020). Less research has been carried out on the wolf population in the Absaroka, and we therefore could not assign scats to individual packs.

### Identification and quantification of prey items

All scats were frozen in a −20°C freezer prior to analysis before being soaked in water for 24 h, and filtered repeatedly through a sieve (0.7–0.5 mm mesh size) using running water. A point-frame was used to randomly select 20 hairs per scat for microscopic examination (Ciucci et al. 2004). Small amounts of hair may become temporarily trapped in the digestive tract of wolves and released at a later time (Ciucci et al. 1996); prey remains that occurred in trace amounts within a single scat (≤ 5%) therefore were not included in the analysis. Hair, bones, teeth, and feathers were identified to the finest taxonomic level possible using identification guides (Williamson 1951; Adorjan and Kolenosky 1969; Moore et al. 1974; Kennedy and Carbyn 1981; De Marinis and Asprea 2006) and known hair samples. We recorded the presence or absence of prey items in each scat. Neonate cervid hair is distinguishable from that of adult cervids from birth until their first molt at 4–5 months of age (Pimlott et al. 1969; Kennedy and Carbyn 1981; De Marinis and Asprea 2006). While we could distinguish neonate from adult cervid hair, we were unable to identify neonate cervids to species. In addition to hair, deciduous teeth (Quimby and Gaab 1957), hooves, and degree of bone ossification were used to classify the age of prey when possible. Because hair identification was the primary means of identifying ungulate prey, only two age categories were possible: ungulates less than 5 months of age were considered neonates, and ungulates older than 5 months of age were considered adults. The hair of mule deer and white-tailed deer are very similar and difficult to discriminate to species; we therefore pooled them together as deer (*Odocoileus* spp.). When adult cervid hair could not be identified to species or genus, it was grouped together in one category (adult cervid). Accuracy of hair identification was assessed by a blind test on hair samples from 30 potential mammalian prey species. All scats were analyzed by one person to reduce potential observer bias (Spaulding et al. 2000).

Relative biomass of prey consumed was approximated using the equation developed by Weaver (1993): X = 0.439 + 0.008Y, where Y represents the average live weight (kg) of a prey species, and X is the estimated biomass (kg) consumed of that particular prey per scat. Prey biomass associated with each scat (X) was multiplied by the number of scats containing that particular prey to estimate the total amount of biomass consumed for each prey category in the sample of scats. We calculated the percent biomass of prey consumed by dividing the total consumed weight of a particular prey by the overall weight of all consumed mammalian prey. Biomass was not calculated for unidentified prey or nonmammalian prey. The average live mass of individual prey was estimated from literature values and modified by age structure information obtained from the scat sample (Supplementary Data SD1). We plotted percent frequency of occurrence of each prey type and the estimated biomass for all the areas combined and separately for each area to give an overview of the summer diet of wolves.

### Statistical analysis

Data exploration was carried out following the steps recommended by Zuur et al. (2010). To determine wolf diet variability across years and packs, we compared those packs sampled within the same year and within the same area, and yearly variation between the same packs. In Teton, we used two packs (Teton pack and Buffalo pack) to analyze differences in diet for each prey item across years, while six packs sampled in the same years were used to explore pack variation. All Yellowstone samples were collected in 2009 and we used these data to compare prey occurrences among three packs. Due to sampling differences among the three areas, we only were able to investigate yearly variation in the Tetons. Scats from Absaroka were not included in the year or pack analysis because of insufficient sampling and lack of knowledge of pack territories. *G*-test and Yates correction for continuity (Sokal and Rohlf 1995) were used to make diet comparisons among packs and years. Raw frequency data were used for all *G*-tests. Fisher’s exact test was used to compare prey items that occurred at low frequencies (< 5). Results were considered statistically significant when *P* ≤ 0.05.

To explore whether wolves in the Absaroka relied on smaller prey than wolves within the national parks, we grouped prey species by size. We categorized adult moose, adult bison, adult elk, and adult cattle, as large prey. Adult mule deer, adult white-tailed deer, neonate cervids, neonate bison, and adult bighorn sheep were classified as medium prey, and beaver, rodents, birds, and hares, as small prey. We used occurrence of prey size categories as the response variable coded as a Bernoulli variable (0 or 1), and applied generalized linear models (GLMs) to explore differences across packs from the different areas. We only used data collected during 2009, because this was the only year when all areas were sampled. In addition, we decided to include scats collected in Absaroka during 2010 to increase the sample size from that area. Data exploration revealed only small dietary differences between 2009 and 2010 in Absaroka. We included pack as a fixed effect and compared occurrence of each prey mass category coded as a Bernoulli variable (0 or 1). We contrasted Yellowstone and Teton packs with Absaroka as the reference level. For each prey mass category, we fit a model containing pack as an explanatory variable, and compared this with a null model containing only the intercept. We compared the models using AIC_c_ model selection using the “model.sel” function in the MuMIn package and selected the most parsimonious model within ΔAIC_c_ < 2 delta from the top model (Harrison et al. 2018; Bartoń 2020). Only variables where the 95% confidence intervals that did not cross zero were considered informative. We checked model fit by plotting the Pearson residuals against each explanatory variable included in the model to look for patterns (Zuur et al. 2016). We also assessed the predictive ability of the models by plotting receiving operating characteristic (ROC curves) and calculating the area under the curve (AUC) for each model using the pROC package (Robin et al. 2011). All data analyses were performed with R version 4.0.3 (R Development Core Team 2020).

## Results

### Frequencies and biomass of prey items

A total of 1,906 wolf scats were collected and analyzed from the Greater Yellowstone Ecosystem, with the majority collected in Teton (*n* = 1,307), followed by Yellowstone (*n* = 453), and Absaroka (*n* = 146; Supplementary Data SD2). The number of prey items ranged from 1 to 4 in each scat, but 90% of all scats contained only one prey item. Ninety-six percent of hair samples were correctly identified in the blind test.

**Fig. 2.**
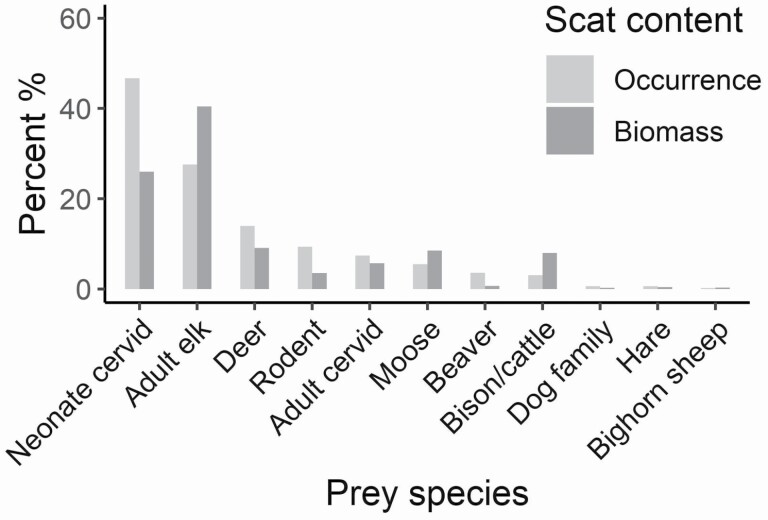
Percent frequency of occurrence and relative biomass of summer prey items for wolf (*Canis lupus*) packs in the Greater Yellowstone Ecosystem (*n* = 1,906 scats, packs = 11, years = 2003–2010).

**Fig. 3.**
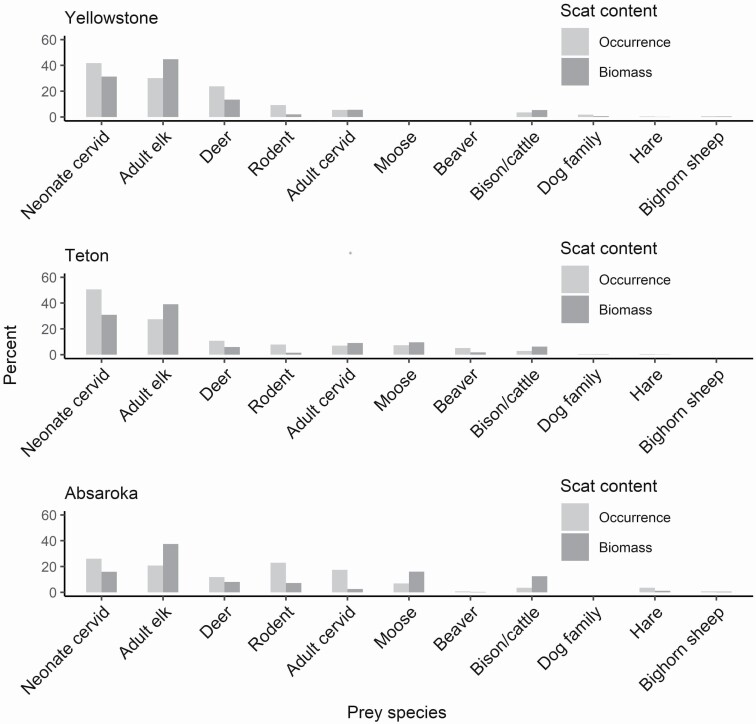
Percent frequency of occurrence and relative biomass of prey items in summer scats of wolf (*Canis lupus*) packs in the Greater Yellowstone Ecosystem. Grand Teton National Park (*n* = 1,307, packs = 8, years = 2003–2009), Yellowstone National Park (*n* = 453, packs = 3, year = 2009), and Absaroka-Beartooth Wilderness (*n* = 146, unknown packs, years = 2009–2010).

Neonate cervids (47%) were the most frequently occurring prey item detected in wolf scats across the Greater Yellowstone Ecosystem, followed by adult elk (28%; Fig. 2). In terms of relative biomass, the trend was opposite, adult elk (40%) contributed a higher relative biomass than neonate cervids (26%). Adult deer and moose represented tertiary prey items (9% biomass, each), along with bison/cattle (8% biomass) and unidentified cervids (6% biomass). Cattle hair was detected in only 3% of scats collected from Absaroka, and was not detected in any scats from Yellowstone or Teton. Small rodents (4% biomass) were the most frequently consumed noncervid prey, whereas other prey items (e.g., beaver, hares, canids, and bighorn sheep) were consumed in negligible amounts (< 1% biomass each; Fig. 2). Neonate cervids, adult elk, and deer were less dominant in the diet of Absaroka wolves, where the diet was more varied across several prey items (Fig. 3). In terms of frequency of occurrence, rodents were among the most common prey species in Absaroka, together with neonate cervids and adult elk. Still, adult elk contributed by far the most to the biomass digested. Wolves consumed moose in Teton and Absaroka (10% and 16% biomass, respectively), but moose was not detected in scats from Yellowstone. Beaver was primarily detected in Teton wolf scats.

### Pack, year, and regional differences

Prey composition derived from scat analysis was compared among years within the same study area in Teton and among packs within the same year in Teton and Yellowstone. Diet varied across years both for the Teton and Buffalo pack (Supplementary Data SD3). Five prey items differed among years for the Teton pack, and three prey items differed among years for the Buffalo pack. The occurrence of neonate cervids and adult moose varied the most across these packs. The occurrence of prey items also varied among packs within the same year of study, with the largest variability in terms of occurrence of neonate cervids, adult elk, and adult moose (Supplementary Data SD4). In years with a high occurrence of neonate cervids, the wolf diet comprised a lower occurrence of other prey species, but in years with a low occurrence of neonate cervids, wolves displayed a more variable diet with higher occurrence of a wider range of prey species.

**Fig. 4.**
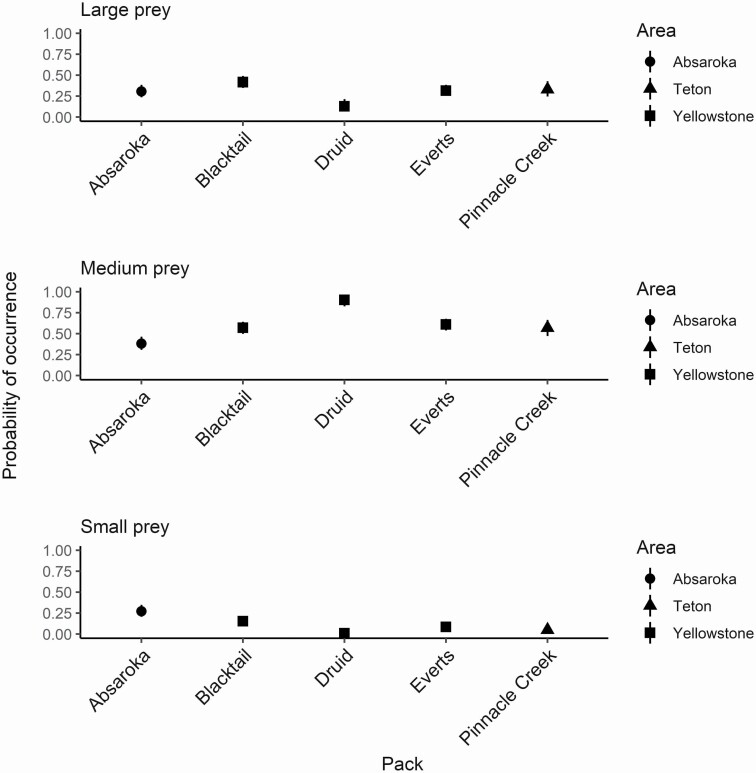
Predicted probability of large (~267–585 kg), medium (~50–70 kg), and small prey (≤ 20 kg) occurrence in the summer diet of wolf (*Canis lupus*) packs between the three areas of the Greater Yellowstone Ecosystem. Grand Teton National Park (*n* = 100, packs = 1, year = 2009), Yellowstone National Park (*n* = 453, packs = 3, year = 2009), and Absaroka-Beartooth Wilderness (*n* = 146, packs unknown, years = 2009–2010). Predictions are based on a generalized linear model with a Bernoulli distribution. The 95% confidence intervals around each estimate are represented by the vertical lines.

All the models contrasting occurrence of different prey mass categories in Absaroka compared to wolf packs in Yellowstone and Teton were found to be important when compared to a null model (large prey ΔAIC_c_: 17.63, medium prey ΔAIC_c_: 63.37, and small prey ΔAIC_c_: 42.31; Supplementary Data SD5). Using Absaroka as the reference level, only two packs were informative and different in terms of occurrence of large prey (Table 1). The Blacktail pack in Yellowstone had a higher probability of large prey in their diet, while the Druid pack in Yellowstone had a lower probability of large prey (Fig. 4). All of the packs were informative when the occurrence of medium and small prey in the wolf diet of packs in Telton and Yellowstone was contrasted against Absaroka. Absaroka wolves had a lower probability of medium prey and higher probability of small prey compared to all packs in the national parks. The predictive power of these models in terms of ROC curves varied with AUC ranging from 0.60 to 0.72 (Supplementary Data SD5 and SD6). The model predicting the occurrence of small prey between the packs from the three areas had the overall highest predictive accuracy of 0.72.

**Table 1.** Estimates, standard error, *z*-value, *P*-value, and 95% confidence interval, of model terms to predict the occurrence of large (~267–585 kg), medium (~50–70 kg), and small prey (≤ 20 kg) categories in the summer diet of wolves (*Canis lupus*) in three areas of the Greater Yellowstone Ecosystem. Grand Teton National Park (*n* = 100, packs = 1, year = 2009), Yellowstone National Park (*n* = 453, packs = 3, year = 2009), and Absaroka-Beartooth Wilderness (*n* = 146, packs unknown, years = 2009–2010).

Large prey variable	Estimate	*SE*	*z*-value	*P*	2.5%	97.5%
Intercept(Absaroka)	−0.82	0.18	−4.54	< 0.001	−1.18	−0.47
PackBlacktail	0.49	0.24	2.05	0.04	0.02	0.96
PackDruid	−1.09	0.36	−3.04	< 0.01	−1.83	−0.41
PackEverts	0.05	0.24	0.19	0.85	−0.42	0.52
PackPinnacle Creek	0.11	0.28	0.40	0.69	−0.44	0.66
Medium prey	Estimate	*SE*	*z*-value	*P*	2.5%	97.5%
Intercept(Absaroka)	−0.48	0.17	−2.81	0.01	−0.82	−0.15
PackBlacktail	0.77	0.23	3.35	< 0.001	0.32	1.22
PackDruid	2.71	0.39	6.95	< 0.001	2.00	3.54
PackEverts	0.93	0.23	4.07	< 0.001	0.48	1.38
PackPinnacle Creek	0.76	0.27	2.88	< 0.01	0.25	1.29
Small prey	Estimate	*SE*	*z*-value	*P*	2.5%	97.5%
Intercept(Absaroka)	−0.99	0.19	−5.28	< 0.001	−1.37	−0.63
PackBlacktail	−0.71	0.28	−2.53	0.01	−1.27	−0.16
PackDruid	−3.53	1.02	−3.45	< 0.001	−6.42	−1.97
PackEverts	−1.38	0.32	−4.29	< 0.001	−2.03	−0.77
PackPinnacle Creek	−1.95	0.50	−3.94	< 0.001	−3.05	−1.07

## Discussion

In accordance with our hypothesis, the summer diet of wolves had a large component of medium-sized prey, including neonate cervids and adult deer, and some smaller sized species. However, large prey such as adult elk contributed the most to their diet in terms of biomass consumed. The Absaroka wolves generally had a larger variation of prey species compared to wolves within the national parks. The difference between packs and yearly variation supports our hypothesis regarding the opportunistic nature of the summer diet of wolves. The diet of wolves was more variable with higher occurrences of several species when cervid neonates were less prevalent in the diet. We partly confirmed our hypothesis concerning differences in summer diet between the three regions. Absaroka wolves had a higher probability of small prey in their diet and a lower probability of medium-sized prey compared to packs in the national parks. We could not confirm that the wolf packs in Yellowstone and Teton consumed more large prey compared to Absaroka.

### Sampling design and data limitations

For this type of research, a uniform sampling design is ideal, but this was not possible in our study because scat was collected as part of independent projects within each area. We pooled data from these three projects to make inferences about the overall summer diet of wolves in the Greater Yellowstone Ecosystem. It is important to note that confounding factors related to scat collection techniques (i.e., homesites versus opportunistic sampling—Steenweg et al. 2015), year, and month, of collection could have confounded the results of our analysis. We have tried to account for the dependency in the data within the same area, year, and packs, but there may exist additional dependencies, for example, between scats sampled in the same locations. However, sampling from high-use areas such as dens and rendezvous sites was necessary to achieve a sufficient sample size. In addition, scats were collected during a longer season in Absaroka and Teton compared to Yellowstone, which may have an impact on the observed regional differences.

Various scat analysis methodologies have been used in carnivore diet studies; each has its limitations, biases, and interpretational difficulties (Kelly 1991; Reynolds and Aebischer 1991; Ciucci et al. 1996, Marucco et al. 2008; Klare et al. 2011). In this study, we used both frequency of occurrence and relative biomass to document wolf diet. According to Klare et al. (2011), the technique most commonly applied in published carnivore diet studies is frequency of occurrence, which is useful for comparisons among studies and to detect rare prey species. However, results based on frequency of occurrence data alone should be interpreted with caution, as their validity is questionable when considering ecological parameters. Small prey have a higher surface to volume ratio and are composed of more hair and bone per mass flesh than larger prey (Weaver 1993). Because hair and bones are the primarily ways to identify mammalian prey in scat, small prey can be overestimated using frequency techniques because the presence of small prey items and large prey items are equally weighted in the analysis (Klare et al. 2011).

Predator-specific biomass models are based on captive feeding trials and the relationship between prey biomass consumed per collectable scat produced. These models have been developed to account for disparities in prey body mass for several carnivore species (Klare et al. 2011), including wolves (Weaver 1993). However, the pitfall of using biomass models is that their accuracy is influenced by multiple variables, including errors associated with more than one prey item per scat, and average prey weight variation. In this study, a positive bias may have occurred because 10% of scats used in the biomass calculations contained more than one prey item. While biomass calculation methods have limitations, they are biologically more meaningful and provide the best approximation to true diets of carnivores (Ciucci et al. 1996; Klare et al. 2011).

Scat collection and analysis provide advantages compared to other methods to study wolf diet. While GPS-telemetry techniques adequately detect large-bodied ungulate prey, they are less successful in detecting medium- and small-bodied prey (Sand et al. 2005, 2008; Webb et al. 2008; Knopff et al. 2010; Palacios and Mech 2011; Trejo 2012). Also, scat analysis is noninvasive, cost-efficient, and well-suited for the study of endangered or elusive carnivores (Ciucci et al. 1996). Wolf scats can be collected opportunistically, allowing for sample collection while engaging in other management- and research-related tasks, which can facilitate long-term studies contrasting wolf diet across regions and different management regimes.

### Frequency and biomass of main prey items

In North America, wolves predominantly prey on ungulates throughout the year (Peterson and Ciucci 2003), large- and medium-sized ungulates such as deer, elk, moose, and caribou (*Rangifer tarandus*) are the most common prey across the three continents where wolves persist (Newsome et al. 2016). During summer, wolves tend to prey more on neonate ungulates (Pimlott et al. 1969; Voigt et al. 1976; Fritts and Mech 1981), and consume a greater diversity of prey than in winter (Ballard et al. 1987; Spaulding et al. 1998; Stahler et al. 2006). Wolves in the Greater Yellowstone Ecosystem predominately preyed on wild ungulates, particularly neonate cervids, adult elk, and adult deer. Other prey items such as moose, bison, rodents, beavers, and hares constituted a more variable component of their diet. Prey availability and vulnerability are important factors influencing wolf predation (Mech and Peterson 2003). A seasonal pulse of neonate cervids becomes available to wolves in the Greater Yellowstone Ecosystem in May and June, and they are particularly susceptible to predation in the initial weeks following birth (Barber-Meyer et al. 2008; Metz et al. 2012). Frequency of occurrence of prey species in scats may overestimate the importance of small prey, which may occur frequently in wolf diet, but overall contribute a low biomass (Weaver 1993). For example, neonate cervids were an important diet item in terms of frequency of occurrence, but the estimated biomass shows they are a less important food source. Despite not contributing to the largest biomass in wolf diet, the high frequency of occurrence on neonate cervids in wolf scats might suggest high kill rates that may impact on ungulate populations (Sand et al. 2008; Metz et al. 2020a). However, as we have not surveyed actual wolf kill sites, it is impossible to distinguish whether this represents predation or scavenging (Metz et al. 2020b). Adult elk contributed most to consumed biomass, which is supported by previous dietary studies from the region (Smith et al. 2004; Stahler et al. 2006; Metz et al. 2020b). However, secondary prey items (e.g., deer, moose, bison, rodents, and hares) may be an important addition to the diet of wolves during certain times of the year, and could sustain wolves between elk kills, which may be particularly important while wolves are tied to homesites while rearing pups.

### Pack and yearly diet variation

Prey composition varied considerably among different packs in Teton and Yellowstone, especially for neonate cervids, elk, and moose, while the occurrence of other prey items was overall low. Packs that had a high occurrence of neonate cervid as prey items often had a lower occurrence of other species, and conversely packs with a low occurrence of neonate cervids had a more variable diet with a higher number of other species. Pack size might impact prey selection, as smaller packs may be limited in their ability to prey on large species (MacNulty et al. 2014); however, we did not consider the effect of pack size due to the low pack cohesiveness during summer (Metz et al. 2011). The differences also may reflect varying local densities of prey species (Metz et al. 2020b). Still, previous studies have found that wolf packs may display very different diets, even within highly homogenous landscapes with constant prey supply (Derbridge et al. 2012). Significant dietary differences have even been found among individuals from the same pack (Stanek et al. 2017), which may be related to the age of the sampled wolf (Gable et al. 2017). We could not explore such individual differences due to our sampling method, making it challenging to relate scats to individual wolves. The observed pack differences therefore could relate both to different feeding habits among individuals or packs as well as to different environmental conditions within the wolf pack territories (Milakovic and Parker 2011). Nelson et al. (2012) found that wolves used different strategies in summer to cope with the decreased availability of elk due to seasonal migratory shifts. Some wolves did not alter their distribution and used alterative prey such as mule deer, while other wolves made extraterritorial forays away from their homesites to access summer elk ranges or shifted the location of rendezvous sites (Nelson et al. 2012).

We also identified yearly variation in the summer diet of wolves in Teton, where scats were sampled from the same pack over several years. This variation illustrates that their diet is not only spatially, but also temporally variable. Occurrence of neonate cervids varied the most among years, followed by adult elk, moose, and bison. In years when neonate cervids were less prevalent in the diet, wolves tended to consume more adult elk and moose. Such yearly variation in wolf diet also has been found in several other studies including Italian wolves switching between wild boar (*Sus scrofa*) and livestock, reflecting potential changes in abundance or access to prey (Ciucci et al. 2018). Stable isotope analysis of wolf scats from British Columbia similarly found variation in the summer diet across years (Milakovic and Parker 2011). Wolf diet even may vary among different weeks within the summer season (Gable et al. 2018a), which we could not account for due to challenges with dating the scats to specific weeks.

### Regional differences in diet composition

The occurrence of different prey size categories varied among regions with a lower probability of medium prey and a higher probability of small prey in Absaroka compared to packs in Yellowstone and Teton. The duration of scat sampling differed among the three collection areas, which could influence some of the observed differences. Scat collected from Yellowstone were more representative of late spring and summer, while wolf scats collected in Teton and Absaroka were more representative of summer and early fall. However, this cannot explain why the diet of packs in both national parks were different from Absaroka. The observed difference also may be related to differing prey abundances among the three study areas (Metz et al. 2012; Rickbeil et al. 2019; Woodruff and Jimenez 2019). In the Absaroka, smaller prey were more prevalent in the diet of wolves compared to the two other study areas. Rodents were the second most commonly occurring prey item in the scat analysis. The high occurrence of small prey in Absaroka compared to the national parks indicates the great flexibility in wolf diet that can enable them to persist in a variety of ecosystems outside pristine national parks, which may have a lower density of large ungulates. Rodents and small mammals also have been found to occur more often in the diet of wolves in the Arctic, and in high-elevation areas (Newsome et al. 2016), which supports our similar findings from the higher elevations found in Absaroka.

Medium-sized prey such as deer were found in high occurrences in scats across all three areas, which suggests they are an important part of the summer diet, as noted in previous studies (Metz et al. 2012). This contrasts with winter diet studies from Yellowstone, where deer were not common (Smith et al. 2004; Metz et al. 2012). Wolves from Yellowstone had the overall highest occurrence of deer in the summer diet, which may be a response to lower elk availability because some elk migrate out of the Northern Range during summer (White et al. 2010). Moose did not occur in the diet of wolves in Yellowstone, probably due to the reduction of moose population since the wildfires of 1988 (Tyers 2003, 2006). However, moose was present in the diets of wolves in Teton and Absaroka, but at a lower proportion of the diet (~10% and 16% of biomass) compared to previous studies in other ecosystems. A study in northern Montana found that moose constituted 18% of the prey biomass consumed (Derbridge et al. 2012), while in Ontario, Canada, traces of moose were found in 51.5% of all scats (Found et al. 2018).

Predation of bighorn sheep occurred only in one pack in Yellowstone and was found in one scat from the Absaroka. Encounters between wolves and bighorn sheep likely are infrequent because of the low population of bighorn sheep in Yellowstone and low habitat overlap with bighorn sheep (Smith et al. 2003). Beaver was detected in wolf scats from Teton and Absaroka, but not in Yellowstone where beavers still are recovering following reintroduction into Absaroka and gradual dispersal into Yellowstone (Smith et al. 2003; McColley et al. 2012; Scrafford et al. 2018). Several studies of wolf diets in North America have shown that beaver is an important prey species during summer (Forbes and Theberge 1996; Tremblay et al. 2001; Gable et al. 2018b), which may suggest that beavers could become a more important food source if the population increase. Overall, smaller prey species may provide an important food source for wolves during summer due to the lower energy requirements during that time, and limited access to other prey while restricted to the den or rendezvous sites (Stahler et al. 2006; Newsome et al. 2016).

We have illustrated how a long-term collection of wolf scats across an entire ecosystem can reveal both spatial and temporal variation in wolf summer diet. We identified a high occurrence of neonate cervids in the diet of the wolves, which may be missed by other research methods that rely on detecting kill sites. Scat analysis using various sampling methods should still be interpreted with caution and future studies should explore the degree to which different sampling regimes can influence wolf diet analysis. The results from this study indicate a highly variable summer diet, with regional, yearly, and pack, differences. Our findings illustrate the importance of considering pack and yearly differences when exploring wolf diets, while it raises new ecological questions necessary to explain what drives these differences. Future studies should explore potential explanatory variables such as local prey abundance, timing of ungulate calving, and social cohesion of wolf packs. The high flexibility of the wolf summer diet illustrates the importance of studying their diet across both spatial and temporal scales to understand wolf summer predation, and wider impact on prey and the ecosystem.

## Supplementary Data

Supplementary data are available at *Journal of Mammalogy* online.

**Supplementary Data SD1.**—Estimated average mass (kg) of prey categories used to calculate relative biomass of prey items.

**Supplementary Data SD2.**—Number of wolf (*Canis lupus*) scats included in the analysis from each of the three areas of the Greater Yellowstone Ecosystem.

**Supplementary Data SD3.**—Statistical significance for differences in wolf diet between years for wolf packs in Grand Teton National Park.

**Supplementary Data SD4.**—Statistical significance for differences in wolf diet between packs in Grand Teton National Park and Yellowstone National Park.

**Supplementary Data SD5.**—Summary of model selection with AIC_c_ values to determine regional difference in the occurrence of large, medium, and small prey categories in the diet of wolf packs between regions of the Greater Yellowstone Ecosystem.

**Supplementary Data SD6.**—Receiver operating characteristic (ROC) curves of the generalized linear model (GLM) predicting the occurrence of large, medium, and small prey items in the diet of wolves (*Canis lupus*) between three regions of the Greater Yellowstone Ecosystem.

gyab060_suppl_Supplementary_Data_SD1

gyab060_suppl_Supplementary_Data_SD2

gyab060_suppl_Supplementary_Data_SD3

gyab060_suppl_Supplementary_Data_SD4

gyab060_suppl_Supplementary_Data_SD5

gyab060_suppl_Supplementary_Data_SD6
