# Supplementary material for: Spatial and temporal variability in summer diet of gray wolves (Canis lupus) in the Greater Yellowstone Ecosystem
Source: J Mammal. 2021 May 29;102(4):1030–41. doi: 10.1093/jmammal/gyab060 (PMC8362331; doi:10.1093/jmammal/gyab060)
Supplement: gyab060_suppl_Supplementary_Data_SD1 [file gyab060_suppl_supplementary_data_sd1.docx]

Supplementary Data SD1. Estimated average mass (kg) of prey categories used to calculate relative biomass consumed by wolves (*Canis lupus*) in the Greater Yellowstone Ecosystem.

| **Prey** | **Live mass (kg)** | **Source** |
| --- | --- | --- |
| Adult elk | 267 | Averaged mass from Houston (1982) and Quimby and Johnson (1951) |
| Neonate elk | 50 | Extrapolated mass from Cook (2002) |
| Adult deer | 67 | Averaged mass from (Anderson et al., 1974) |
| Neonate deer | 13 | Averaged mass from (Anderson, 1981) and (Pojar and Bowden, 2004) |
| Adult moose | 358 | Averaged mass from Blood et al. (1967), Schladweiler and Stevens (1973),  and Franzmann et al. (1978) |
| Neonate moose | 60 | Averaged mass from Blood et al. (1967) and (Coady, 1973) |
| Undetermined  adult cervid | 167 or 230 | Yellowstone: averaged mass of adult elk and adult deer was 167 kg. Grand Teton: average mass of adult elk, adult deer, and adult moose was 230 kg. |
| Adult bison | 585 | Averaged mass from Berger and Peacock (1988) |
| Adult bighorn sheep | 70 | Averaged mass from Burt and Grossenheider (1980) |
| Beaver | 20 | Averaged mass from Burt and Grossenheider (1980) |
| Small rodents | 1 | Averaged mass of four rodent species^a^ from Burt and Grossenheider (1980) |
| Lagomorphs | 2 | Averaged mass of four lagomomorph species^b^ from Burt and Grossenheider (1980) |
| ^a^ *Marmota flaviventris, Urocitellus armatus, Thomomys talpoides,* and *Microtus pennsylvanicus* | | |
| ^b^ *Lepus americanus, Lepus townsendii, Sylvilagus audubonii*, and *Sylvilagus nuttallii* | | |

**LITERATURE CITED**

Anderson, A. E., 1981. Morphological and physical condition, in Mule and black-tailed deer of North America (O. C. Wallmo, eds). University of Nebraska, Lincoln, Nebraska.

Anderson, A. E., D. E. Medin, D. C. Bowden. 1974. Growth and morphometry of the carcass, selected bones, organs, and glands of mule deer. Wildlife Monographs 39:3-122.

Berger, J., M. Peacock, 1988. Variability in Size-Weight Relationships of *Bison bison*. Journal of Mammalogy 69:618-624.

Blood, D., J. R. McGillis, A. L. Lovaas, 1967. Weights and measurements of moose in Elk Island National Park, Alberta. Canadian Field Naturalist 81:263-269.

Burt, W. H., R. P. Grossenheider, 1980. Peterson field guide to mammals. Houghton Mifflin Company. Boston, Massachusett.

Coady, J. W., 1973. Interior moose studies. Federal Aid Wildlife Restoration Project. Progress Report W-17-4 and W-17-5, Alaska Department of Fish and Game, Juneau, Alaska.

Cook, J. G., 2002. Nutrition and food in North American elk: ecology and management (Toweill, D. E., J. W. Thomas, eds). Smithsonian Institution, Washington D.C.

Franzmann, A. W., R. E. LeResche, R. A. Rausch, J. L. Oldemeyer, 1978. Alaskan moose measurements and weights and measurement-weight relationships. Canadian Journal of Zoology 56:298-306.

Houston, D., 1982. The northern Yellowstone elk: ecology and management. Macmillan New York, USA.

Pojar, T. M., D. C. Bowden, 2004. Neonatal mule deer fawn survival in west-central Colorado. Journal of Wildlife Management 68:550-560.

Quimby, D. C., D. E. Johnson, 1951. Weights and measurements of Rocky Mountain elk. The Journal of Wildlife Management 15:57-62.

Schladweiler, P., D. R. Stevens, 1973. Reproduction of Shiras moose in Montana. The Journal of Wildlife Management 37:535-544.
