# Supplementary material for: Spatial and temporal variability in summer diet of gray wolves (Canis lupus) in the Greater Yellowstone Ecosystem
Source: J Mammal. 2021 May 29;102(4):1030–41. doi: 10.1093/jmammal/gyab060 (PMC8362331; doi:10.1093/jmammal/gyab060)
Supplement: gyab060_suppl_Supplementary_Data_SD2 [file gyab060_suppl_supplementary_data_sd2.docx]

Supplementary Data SD2: Number of wolf (*Canis lupus*) scats included in the analysis from each of the three areas of the Greater Yellowstone Ecosystem (n= 1906 scats, packs=11, years= 2003 –2010) with description of year, pack (if known), and scat collection sites or technique.

| Location | Pack | Year | Scat collection site/technique | *n* | *Total scats* |
| --- | --- | --- | --- | --- | --- |
| Yellowstone National Park | Mount Everts | 2009 | Den, Rendezvous, and opportunistically collected | 185 | 453 |
|  | Blacktail | 2009 | Den, Rendezvous, and opportunistically collected | 175 |  |
|  | Druid | 2009 | Den | 93 |  |
|  |  |  |  |  |  |
| Grand Teton National Park  (and the National Elk Refuge) | Teton | 2003 | Den | 102 | 1307 |
|  |  | 2004 | Den | 97 |  |
|  |  | 2005 | Den, Rendezvous | 186 |  |
|  | Flat Creek | 2005 | Rendezvous | 154 |  |
|  | Buffalo | 2006 | Den | 82 |  |
|  |  | 2007 | Den | 203 |  |
|  |  | 2008 | Den | 79 |  |
|  | Pacific Creek | 2006 | Den | 117 |  |
|  | Huckleberry | 2007 | Den | 43 |  |
|  | Antelope | 2008 | Den | 36 |  |
|  | Phantom Springs | 2008 | Den | 108 |  |
|  | Pinnacle Peak | 2009 | Den | 100 |  |
|  |  |  |  |  |  |
| Absaroka Beartooth Wilderness | NA | 2009 | Transect line | 59 | 146 |
|  | NA | 2010 | Transect line | 87 |  |
|  |  |  | Grand Total | | 1906 |
