# Supplementary material for: Spatial and temporal variability in summer diet of gray wolves (Canis lupus) in the Greater Yellowstone Ecosystem
Source: J Mammal. 2021 May 29;102(4):1030–41. doi: 10.1093/jmammal/gyab060 (PMC8362331; doi:10.1093/jmammal/gyab060)
Supplement: gyab060_suppl_Supplementary_Data_SD3 [file gyab060_suppl_supplementary_data_sd3.docx]

Supplementary Data SD3: Differences in summer wolf (*Canis lupus*) diet between years for the Teton pack (n= 385) and the Buffalo pack (n= 364) in Grand Teton National Park. G-test or Fisher’s Exact test were used for each comparison and the G-test statistic and P-value is provided. Differences were considered significant at a significance level of 0.05.

| Pack (years) | Prey item | Test | G-test statistic | P-value |
| --- | --- | --- | --- | --- |
| Teton (2003 –2005) | Neonate cervid | G-test | 23.45 | < 0.01 |
|  | Adult elk | G-test | 11.37 | < 0.01 |
|  | Adult deer | G-test | 6.93 | 0.03 |
|  | Adult moose | G-test | 12.26 | < 0.01 |
|  | Beaver | Fisher's Exact | - | 0.25 |
|  | Small rodents | G-test | 6.68 | 0.04 |
|  |  |  |  |  |
| Buffalo (2006 –2008) | Neonate cervid | G-test | 31.97 | < 0.01 |
|  | Adult elk | G-test | 3.54 | 0.17 |
|  | Adult deer | Fisher's Exact | - | 0.14 |
|  | Adult moose | G-test | 45.94 | < 0.01 |
|  | Bison (all ages) | Fisher's Exact | - | 0.01 |
|  | Beaver | G-test | 2.56 | 0.28 |
|  | Small rodents | G-test | 1.1 | 0.58 |
|  | Lagamorph | Fisher's Exact | - | 1.00 |
