# Supplementary material for: Spatial and temporal variability in summer diet of gray wolves (Canis lupus) in the Greater Yellowstone Ecosystem
Source: J Mammal. 2021 May 29;102(4):1030–41. doi: 10.1093/jmammal/gyab060 (PMC8362331; doi:10.1093/jmammal/gyab060)
Supplement: gyab060_suppl_Supplementary_Data_SD4 [file gyab060_suppl_supplementary_data_sd4.docx]

Supplementary Data SD4: Differences in summer wolf (*Canis lupus*) diet between packs in Grand Teton National Park and Yellowstone National Park. G-test or Fisher’s Exact test were used for each comparison and the G-test statistic and P-value is provided.

| Pack comparison (year) | Prey item | Test | G-test  statistic | P-value |
| --- | --- | --- | --- | --- |
| Teton (n=186)  vs.  Flat Creek (n=154)  (2005) | Neonate cervid | G-test | 22.20 | < 0.01 |
|  | Adult elk | G-test | 24.69 | < 0.01 |
|  | Adult deer | G-test | 12.90 | < 0.01 |
|  | Small rodents | G-test | 18.23 | < 0.01 |
|  |  |  |  |  |
| Buffalo (n=82)  vs.  Pacific Creek (n=117) (2006) | Neonate cervid | G-test | 20.57 | < 0.01 |
|  | Adult elk | G-test | 0.00 | 1.00 |
|  | Adult deer | G-test | 0.02 | 0.88 |
|  | Adult moose | G-test | 20.30 | < 0.01 |
|  | Beaver | G-test | 0.06 | 0.80 |
|  | Small rodents | G-test | 0.51 | 0.48 |
|  |  |  |  |  |
| Buffalo (n=79)  vs.  Phantom Spring (n=108) (2008) | Neonate cervid | G-test | 68.44 | < 0.01 |
|  | Adult elk | G-test | 35.93 | < 0.01 |
|  | Adult deer | G-test | 0.65 | 0.42 |
|  | Adult moose | Fisher's Exact | - | 0.01 |
|  | Small rodents | G-test | 6.29 | 0.01 |
|  |  |  |  |  |
| Blacktail (n=175) vs.  Mount Everts (n=185)  vs.  Druid (n=93) (2009) | Neonate cervid | G-test | 23.75 | < 0.01 |
|  | Adult elk | G-test | 110.02 | < 0.01 |
|  | Adult deer | G-test | 38.56 | < 0.01 |
|  | Bison (all ages) | Fisher's Exact | - | < 0.01 |
|  | Small rodent | G-test | 17.50 | < 0.01 |
