# Supplementary material for: Spatial and temporal variability in summer diet of gray wolves (Canis lupus) in the Greater Yellowstone Ecosystem
Source: J Mammal. 2021 May 29;102(4):1030–41. doi: 10.1093/jmammal/gyab060 (PMC8362331; doi:10.1093/jmammal/gyab060)
Supplement: gyab060_suppl_Supplementary_Data_SD5 [file gyab060_suppl_supplementary_data_sd5.docx]

Supplementary Data S5: ΔAIC_c_ AIC weights and area under the curve (AUC) for GLM models containing pack as a fixed effect contrasted with a null model. The pack effect explores variation in diet between wolf (*Canis lupus*) packs within three areas of the Greater Yellowstone Ecosystem; Grand Teton National Park (n=100, packs: 1, year: 2009), Yellowstone National Park (n= 455, packs= 3, year= 2009) and Absaroka-Beartooth Wilderness (n=144, packs unknown, years= 2009 –2010). The response variable is the occurrence large (~ 267 –585 kg), medium (~ 50 –70 kg) and small prey (≤ 20 kg) items in wolf summer diet of wolves coded as a Bernoulli variable (n= 699).

| Large prey |  |  |  |  |  |  |
| --- | --- | --- | --- | --- | --- | --- |
| Fixed effects | df | logLik | AIC_c_ | ΔAIC_c_ | weight | AUC |
| Area | 5 | -423.28 | 856.6 | 0 | 1 | 0.6 |
| ~ 1 | 1 | -436.14 | 874.3 | 17.63 | 0 |  |
| Medium prey |  |  |  |  |  |  |
| Fixed effects | df | logLik | AIC_c_ | ΔAIC_c_ | weight | AUC |
| Area | 5 | -438.26 | 886.6 | 0 | 1 | 0.65 |
| ~ 1 | 1 | -473.98 | 950 | 63.37 | 0 |  |
| Small prey |  |  |  |  |  |  |
| Fixed effects | df | logLik | AIC_c_ | ΔAIC_c_ | weight | AUC |
| Area | 5 | -239.38 | 488.8 | 0 | 1 | 0.72 |
| ~ 1 | 1 | -264.58 | 531.2 | 42.31 | 0 |  |
