# Supplementary material for: Spatial and temporal variability in summer diet of gray wolves (Canis lupus) in the Greater Yellowstone Ecosystem
Source: J Mammal. 2021 May 29;102(4):1030–41. doi: 10.1093/jmammal/gyab060 (PMC8362331; doi:10.1093/jmammal/gyab060)
Supplement: gyab060_suppl_Supplementary_Data_SD6 [file gyab060_suppl_supplementary_data_sd6.docx]

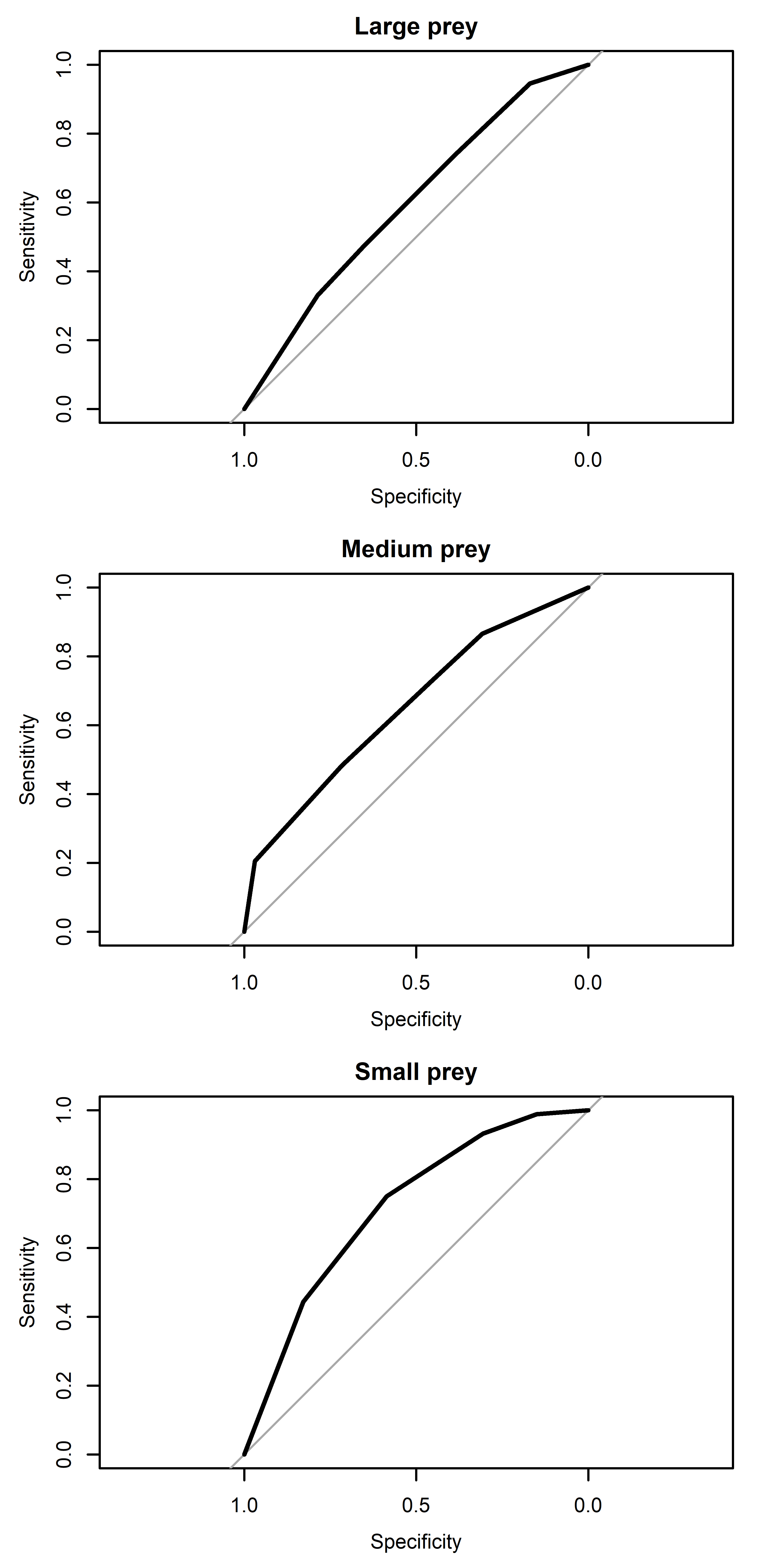


Supplementary Data SD6: Receiver operating characteristic (ROC) curves of the GLM model predicting the occurrence of large, medium and small prey items in the diet of wolves (*Canis lupus*) between three areas of the Grand Teton National Park (n= 100, packs= 1, year= 2009), Yellowstone National Park (n= 455, packs= 3, year= 2009) and Absaroka-Beartooth Wilderness (n= 144, packs unknown, years= 2009­ –2010). The response variable is the occurrence large (~ 267 –585 kg), medium (~ 50 – 70 kg) and small (≤ 20 kg) items in wolf summer diet of wolves coded as a Bernoulli variable (n=699).
